# Association between delayed initiation of treatment indications and survival in patients with cervical cancer: A systematic review and meta-analysis protocol

**DOI:** 10.1371/journal.pone.0271604

**Published:** 2022-07-20

**Authors:** Tariku Shimels, Biruck Gashaw, Teferi Gedif

**Affiliations:** 1 Research Directorate, Saint Paul’s Hospital Millennium Medical College, Addis Ababa, Ethiopia; 2 Gynecologist and Obstetrician, Saint Paul’s Hospital Millennium Medical College, Addis Ababa, Ethiopia; 3 Social and Administrative Pharmacy, School of Pharmacy, College of Health Sciences, Addis Ababa University, Addis Ababa, Ethiopia; Elizabeth Glaser Pediatric AIDS Foundation, UNITED REPUBLIC OF TANZANIA

## Abstract

**Background:**

Cervical cancer is a growing public health problem globally. Despite the availability of management options, the progression of the disease as a function of waiting time may challenge the effort to attain a desired outcome. There is a conflicting report on the role of waiting time to initiate an appropriate treatment in improving patients’ survival.

**Objective:**

This review aims to evaluate the association between delayed time to initiate any treatment indication with survival in patients with cervical cancer.

**Methods:**

An internet-based literature search will be performed using text words, MESH terms and truncated words in databases, namely MEDLINE, Cochrane CENTRAL, EMBASE, Web of Science and Scopus. Grey literature searches in Google Scholar, Networked Digital Library of Theses and Dissertations (NDLTD) and Dissertations and Theses Global will be made. All articles published until 30^th^ of December 2021 on human subjects will be searched without a language restriction. Studies which fulfil the inclusion criteria will be screened in full reading, selected, appraised and assessed for methodological quality by two independent reviewers. Data on participants, study methods, interventions, and outcomes will be abstracted. Included studies will be pooled for meta-analysis. Microsoft-Excel and R packages will be employed to carry out the statistical analysis. Heterogeneity will be assessed using Cochrane Q statistic, Tau^2^, and I^2^. Results will be reported as a function of 4-week delay in treatment initiation and the corresponding hazard ratio (HR) at 95% confidence interval. Statistical significance will be considered at P<0.05.

**Trial registration:**

**PROSPERO registration number:** CRD42022299689.

## Background

Cervical cancer is a malignant tumor of the cervix that emerges at the junction between the outer squamous cell layer and inner columnar cell lining of the cervix [1]. The World Health Organization (WHO) classifies cervical cancer histologically into the following subtypes, squamous cell carcinoma, adenocarcinoma, clear cell adenocarcinoma, adeno-squamous carcinoma, serous carcinoma, glassy cell carcinoma, adenoid basal carcinoma, adenoid cystic carcinoma, undifferentiated carcinoma, and small cell carcinoma [2]. The most common subtypes are squamous cell carcinoma, comprising of nearly 75% of cases [3], followed by adenocarcinoma with10% to 25% of cases [4].

Cervical cancer remains to be the fourth common type cancer in women worldwide [5, 6]. The disease also covers about 22.2% of all cancers in this population of the Sub-Saharan Africa which also puts it as the top most cause of death in the region [7]. In Ethiopia, cervical carcinoma ranks the fourth most common type of cancer in women [8] which also accounts for the second most deadly cancer among this population [9].

Treatment indications for cervical cancer encompasses a concurrent or sequential chemotherapy and radiotherapy, or surgery [10]. Radiotherapy and radical surgery are shown to be effective for managing locally advanced cervical cancer (stages IB, IIA), and that the effectiveness decreases with tumor size [11]. The addition of chemotherapeutic agents, such as cisplatin, carboplatin, and hydroxyurea to definitive irradiation, or treatment of patients with concomitant chemoradiotherapy has also produced a proven superior outcome compared to radiotherapy alone [12], or chemotherapy alone [13].

Both timely screening (diagnosis) and treatment initiation are recommended for early control of progression of cervical cancer. Time from diagnosis to initiation of treatment is one of the several factors associated with survival of patients with cervical cancer [14, 15]. Evidence shows delayed time to treatment initiation significantly increases the risks of mortality in this population [15–17]. Nonetheless, a report on the invariance of progression risk among those at stage I patients with delayed and early surgery wait time exists. Hitherto, the precise direction and association and effect of this relationship are not clearly understood.

This review aims to pool all available literature, and present a timely update on the association between delayed initiation of treatment and survival time in patients with cervical cancer.

## Methods

### Search strategy

The search strategy will employ the use of keywords, index/mesh terms, truncated words, and references of other studies to ensure a maximum possibility in including all eligible articles. Search will be facilitated using the Boolean operators (AND/and OR). Bibliographic searches in MEDLINE, EMBASE, Cochrane CENTRAL, CINHAL, Scopus, and Web of science will be performed. Similarly, grey literature sources, such as Google Scholar and Networked Digital Library of Theses and Dissertations (NDLTD) and Dissertations and Theses Global will be searched. Both English and non-English based articles published earlier to 31^st^ December 2021 will be considered. A supplementary search strategy from MEDLINE has been attached as part of this submission (S1 Appendix).

### Eligibility and exclusion criteria of included studies

All available observational studies (cross-sectional, case-control, cohort) and interventional designs (if available and appropriate to do so) will be considered. The eligibility of studies and populations into the review will take a consideration of the following criteria. 1) All patients diagnosed and histologically confirmed with primary cervical cancer of any stage, and were treated with adjuvant chemotherapy, surgery, and chemoradiation either as concurrent or sequential treatment indication. 2) The time frame between diagnosis, treatment initiation and outcomes of any form (either overall survival or disease free survival) was reported appropriately. 3) Studies analyzed with clear comparator groups. And, 4) studies that reported the effect of delayed treatment initiation adjusted for other prognostic factors. In the contrary, studies for which estimation of the outcome is not in the form of a hazard ratio (HR) or findings reported in other measures of effect size (risk ratio, odds ratio) will be excluded.

### Study screening and assessment of methodological quality

Potential studies collated from all sources via a citation manager (Endnote) will be screened based on abstract and title. After deduplication, evaluation will be carried out in Rayyan web-based application [18]. The studies which fulfilled the inclusion criteria will be assessed in full reading and, sequentially, be recruited for further methodological evaluation. The Newcastle-Ottawa risk of bias assessment tool [19] will be employed to rate the quality of included studies. The reports will be prepared following the steps in the ‘meta-analysis of observational studies in Epidemiology(MOOSE)’ [20].

### Data extraction and procedure

Studies screened at a title and abstract level will be read in full text before actual data extraction commences. For studies with no full text or non-English substantial data, authors will be contacted via email. Three reviewers will be involved to accomplish the screening, quality assessment, and data extraction processes. Whereas, two of the reviewers (TS and BG) will involve independently, a third reviewer (TG) will be consulted at times if disputing results rise between the primary reviewers. Data on study designs, quality assessment, authors and year of publication, overall survival (OS), and disease free survival (DFS) either as hazard ratios (HRs) or an appropriate measure of effect that can, latter, be converted to a HR and its 95% confidence interval, number of patients evaluated, waiting times, study design, age, cancer stage, and factors adjusted will be extracted.

### Exposure and outcome measures

Exposure will be the delayed or waiting time to initiate any recommended treatment. The delayed time considered for this review will be a 4-week waiting time computed from the reported exposure time in each study. For studies with more than two categories, the midpoint/median time of the respective class will be considered an exposure level as appropriate. Pooled coefficients (ßs) will be converted to HRs as a function of 4-week delay in treatment initiation. The study specific hazard ratios, either on OS or DFS, along with their 95% confidence interval will be considered as outcomes of interest. The terms ‘disease free survival, recurrence free survival, relapse free survival’ will be treated as disease free survival (DFS) as also used in elsewhere [21]. The log [HRs] will be divided by the number of weeks in each study to estimate study specific coefficients (ßs) that will be imputed in the pooled HR. The coefficient in studies with more than two waiting time categories will be computed using meta-regression.

### Meta-analysis

A quantitative data synthesis will be performed in Microsoft-Excel and R package. When necessary, we will manipulate the data in Statistical Packages for Social Sciences (SPSS v.26, Armonk, NY, IBM Corp.) [22], and transform into the R package for analysis [23]. Study-based ßs will be computed from hazard ratios [HRs] reported. Pooled betas will be converted to HRs as a function of 4-week delay in treatment initiation. Either of a random or fixed effect measures will be considered to model studies depending on the observed heterogeneity. Q statistic, Tau^2^ and I^2^ will be used to calculate heterogeneity. A subgroup analysis will also be carried out based on heterogeneity of studies and the type of treatment indication patients received, stage of the cancer, and follow-up period. A sensitivity analysis will be carried out by excluding studies that have high influence on the observed heterogeneity. Publication bias will be assessed using funnel plot, and significance of the asymmetry will be tested using Egger’s test.

## Supporting information

S1 Checklist PRISMA-P (Preferred Reporting Items for Systematic review and Meta-Analysis Protocols) 2015 checklist: Recommended items to address in a systematic review protocol*. (DOC)

S1 Appendix Search strategy (MEDLINE). (DOCX)
