# Supplementary material for: Association between delayed initiation of treatment indications and survival in patients with cervical cancer: A systematic review and meta-analysis protocol
Source: PLoS One. 2022 Jul 20;17(7):e0271604. doi: 10.1371/journal.pone.0271604 (PMC9299293; doi:10.1371/journal.pone.0271604)
Supplement: S1 Checklist — (DOC) [file pone.0271604.s001.doc]

**PRISMA-P (Preferred Reporting Items for Systematic review and Meta-Analysis Protocols) 2015 checklist: recommended items to address in a systematic review protocol***

| Section and topic | Item No | Checklist item |
| --- | --- | --- |
| ADMINISTRATIVE INFORMATION | | |
| Title: |  | **Association between delayed treatment initiation and survival in cervical cancer: A systematic review & meta-analysis protocol** |
|  |  |  |
| Registration | 2 | Prospero registration number: CRD42022299689 |
| Authors:  Contact | 3a | Tariku Shimels^1^, Biruck Gashaw^2^, and Teferi Gedif^3^  ^1^Research advisor and coordinator, Research Directorate, Saint Paul’s Hospital Millennium Medical College, Addis Ababa, Ethiopia  Email address: tarphar2008@gmail.com  ^2^Gynecologist and Obstetrician, Saint Paul’s Hospital Millennium Medical College, Addis Ababa, Ethiopia  Email: biruckgashawbeza@gmail.com  ^3^Professor of Social and Administrative Pharmacy, School of Pharmacy, College of Health Sciences, Addis Ababa University, Addis Ababa, Ethiopia  Email address: tgedif@gmail.com |
|  |  |  |
| Contributions | 3b | TS and TG conceptualized the review. BG reviewed the draft protocol. TS developed the protocol write-up. All reviewers have read and approved the final review protocol. |
| Amendments | 4 | N/A. |
| Support: | None. |  |
| Sources | 5a | N/A |
| Sponsor | 5b | N/A |
| Role of sponsor or funder | 5c | N/A |
| INTRODUCTION | | |
| Rationale | 6 | Cervical cancer is a growing public health problem globally. Despite the availability of management option , the progression of the disease as a function of waiting time may challenge the effort to attain a desired success. There is a conflicting report on the role of waiting time to initiate an appropriate treatment in improving patients’ survival outcome. |
| Objectives | 7 | This review aimed to evaluate the association between the delayed times to initiate any treatment indication with survival in patients with cervical cancer. |
| METHODS | | |
| Eligibility criteria | 8 | All available observational studies (cross-sectional, case-control, cohort) and interventional designs (if available and appropriate to do so) will be considered. The eligibility of studies and populations into the review will basically take a consideration of the following criteria. 1) All patients diagnosed and histologically confirmed with primary cervical cancer of any stage, and were treated with adjuvant chemotherapy, surgery, and chemoradiation either as concurrent or sequential treatment indication. 2) The time frame between diagnosis, treatment initiation and outcomes of any form (either overall survival or disease free survival) was reported appropriately. 3) Studies rated as 'good quality' along with the following criteria will be included; a) studies analyzed with clear comparator groups, and b) studies that reported the effect of delayed treatment initiation adjusted for other prognostic factors. In the contrary, studies for which estimation of the outcome in the form of a hazard ratio (HR) or findings reported in other measures of effect size (risk ratio, odds) will be excluded. |
| Information sources | 9 | Studies eligible for this review will be sourced from online databases, reference searching, and grey literature sources. |
| Search strategy | 10 | The search strategy will employ the use of keywords, index/mesh terms, truncated words, and references of other studies to ensure a maximum possibility in including all eligible articles. Search will be facilitated using the Boolean operators (AND/and OR). Bibliographic searches in PubMed, EMBASE, Cochrane CENTRAL, CINHAL, Scopus, and Web of science will be performed. Similarly, grey literature sources, such as Google Scholar and Networked Digital Library of Theses and Dissertations (NDLTD) and Dissertations and Theses Global will be searched. Both English and non-English based articles published earlier to December 2021 will be considered. |
| Study records: |  |  |
| Data management | 11a | Data will be managed both manually and electronically. Electronica data retrieved from online sources will be documented in word and pdf formats. |
| Selection process | 11b | Following the search, all identified citations will be collated and uploaded into EndNote and duplicates will be removed. Next, evaluation will be carried out in Rayyan web based application. The studies which fulfilled the screening keywords will, sequentially, be passed for further methodological evaluation. The Newcastle-Ottawa risk of bias assessment tool will be employed to rate the quality of included studies. The reports will be prepared following the steps in the ‘meta-analysis of observational studies in Epidemiology (MOOSE)’. |
| Data collection process | 11c | Studies screened at a title and abstract level will be read in full text before actual data extraction commences. For studies with no full text or non-English substantial data, authors will be contacted via email. Three reviewers will be involved to accomplish the screening, quality assessment, and data extraction processes. Whereas, two of the reviewers will involve independently, a third reviewer will be consulted at times if disputing results rise between the primary reviewers. |
| Data items | 12 | Data on study designs, quality assessment, authors and year of publication, overall survival (OS), and disease free survival (DFS) either as hazard ratios (HRs) or an appropriate measure of effect that can, latter, be converted to a HR and its 95% confidence interval, number of patients evaluated, waiting times, study design, age, cancer stage, and factors adjusted will be extracted. |
| Outcomes and prioritization | 13 | The study specific hazard ratios, either on OS or DFS, along with their 95% confidence interval will be considered as outcomes of interest. |
| Risk of bias in individual studies | 14 | Eligible studies will be critically appraised by two independent reviewers at the study level for methodological quality in the review using the Newcastle-Ottawa risk of bias assessment scale for observational studies. |
| Data synthesis | 15a | Studies will, where possible, be pooled in statistical meta-analysis using Microsoft Excel, R package, and review manager (RevMan) software version 5.3 as appropriate. |
|  | 15b | Effect sizes will be expressed as either hazard ratio (HR) and the corresponding 95% confidence intervals. Heterogeneity will be assessed statistically using the standard chi-squared, tau squared, and I squared tests. Statistical analyses will be performed using either of fixed or random effect models. |
|  | 15c | A sensitivity analysis will be conducted by excluding certain studies with relative small or huge effect or exclusion of assumptions for missed data (if available). A funnel plot will be generated using RevMan software to assess publication bias. Statistical tests for funnel plot asymmetry will be performed using Egger test. |
|  | 15d | If quantitative synthesis will not appropriate for all or some, data obtained from studies will be interpreted qualitatively. |
| Meta-bias(es) | 16 | The review will assess for any potential biases of selective reporting and publication biases if available. Likely, it will consider and report on any form of biases and whether indirect outcome measures have been used in the analysis instead of pre-specified measurements in the protocol. |
| Confidence in cumulative evidence | 17 | The Grading of Recommendations, Assessment, Development and Evaluation (GRADE) approach for grading the certainty of evidence will be followed and a Summary of Findings (SoF) will be created using GRADEPro GDT 2015. |

*** It is strongly recommended that this checklist be read in conjunction with the PRISMA-P Explanation and Elaboration (cite when available) for important clarification on the items. Amendments to a review protocol should be tracked and dated. The copyright for PRISMA-P (including checklist) is held by the PRISMA-P Group and is distributed under a Creative Commons Attribution Licence 4.0.**

*From: Shamseer L, Moher D, Clarke M, Ghersi D, Liberati A, Petticrew M, Shekelle P, Stewart L, PRISMA-P Group. Preferred reporting items for systematic review and meta-analysis protocols (PRISMA-P) 2015: elaboration and explanation. BMJ. 2015 Jan 2;349(jan02 1):g7647.*
