# Supplementary material for: Association between delayed initiation of treatment indications and survival in patients with cervical cancer: A systematic review and meta-analysis protocol
Source: PLoS One. 2022 Jul 20;17(7):e0271604. doi: 10.1371/journal.pone.0271604 (PMC9299293; doi:10.1371/journal.pone.0271604)
Supplement: S1 Appendix — (DOCX) [file pone.0271604.s002.docx]

**Appendix I: Search strategy (MEDLINE)**

**Table 1**: Keyword and MESH term based search strategy of MEDLINE for observational studies on ‘Association between delayed initiation of treatment indications and survival in patients with cervical cancer: a systematic review and meta-analysis’

| Population | Exposure/Intervention | Outcome |
| --- | --- | --- |
| Patients with histologically confirmed cervical cancer | Delayed ‘’time to initiation of treatment’’ indication | Survival/risk of morality/ recurrence measured as a HR |
| ((((((((cervical cancer) OR (cervical carcinoma)) OR (cervical ca)) OR (cervical ca*)) OR (cancer of the cervix)) AND (patients)) OR (clients)) OR (subjects)) OR (women) | ((((((((((((((((delayed time) OR (time treatment)) OR (time to treatment initiation)) OR (time diagnosis treatment)) OR (time starting treatment)) OR (time first treatment)) OR (delayed initiation radiotherapy)) OR (delayed initiation chemoradiotherapy)) OR (delayed initiation surgery)) OR (time diagnosis surgery)) OR (time diagnosis radiotherapy)) OR (time chemoradiation)) OR (time diagnosis chemotherapy)) OR (time diagnosis adjuvant chemotherapy)) OR (time diagnosis neoadjuvant chemotherapy)) OR (delayed initiation neoadjuvant chemotherapy)) OR (delayed initiation adjuvant chemotherapy) | ((((((((((((((cancer survival) OR (survival))) OR (risk of mortality)) OR (risk of death)) OR (disease free survival)) OR (relapse free survival)) OR (outcome)) OR (mortality)) OR (cancer mortality)) OR (cancer mortality)) OR (cause specific mortality)) OR (hazard)) OR (hazard ratio)) OR (risk of hazard) |
| ("Cervix Uteri"[Mesh] AND "Squamous Intraepithelial Lesions of the Cervix"[Mesh] AND "Atypical Squamous Cells of the Cervix"[Mesh]) AND ("Carcinoma"[Mesh]) | (((((("Time"[Mesh]) OR "Time-to-Treatment"[Mesh]) AND "Radiotherapy, Adjuvant"[Mesh]) OR ( "Drug Therapy"[Mesh] OR "drug therapy" [Subheading] OR "Chemotherapy, Adjuvant"[Mesh] OR "Maintenance Chemotherapy"[Mesh] OR "Chemotherapy, Cancer, Regional Perfusion"[Mesh] OR "Antineoplastic Combined Chemotherapy Protocols"[Mesh] OR "Antineoplastic Agents"[Mesh] OR "Neoadjuvant Therapy"[Mesh] OR "Drug Therapy, Combination"[Mesh] )) OR "Chemoradiotherapy"[Mesh]) OR ( "General Surgery"[Mesh] OR "Surgical Procedures, Operative"[Mesh] OR "surgery" [Subheading] )) OR ( "Therapeutics"[Mesh] OR "therapy" [Subheading] ) Sort by: Most Recent | ((("Fatal Outcome"[Mesh] AND "Treatment Outcome"[Mesh] AND "mortality" [Subheading] AND "Outcome Assessment, Health Care"[Mesh]) OR ( "Mortality"[Mesh] OR "Mortality, Premature"[Mesh] OR "Hospital Mortality"[Mesh] OR "Maternal Mortality"[Mesh] )) OR ( "Survival"[Mesh] OR "Disease-Free Survival"[Mesh] OR "Survival Rate"[Mesh] OR "Progression-Free Survival"[Mesh] OR "Kaplan-Meier Estimate"[Mesh] )) OR ( "Survivors"[Mesh] OR "Survivorship"[Mesh] OR "Cancer Survivors"[Mesh] ) Sort by: Most Recent |
